# Increased Risk of Medical and Implant-Related Complications in Patients With Atrial Fibrillation After Primary Total Shoulder Arthroplasty: A 1:1 Propensity-Matched Cohort Analysis

**DOI:** 10.7759/cureus.106133

**Published:** 2026-03-30

**Authors:** Nishanth Muthusamy, Adrien Canery, Liam Edwards-Gaherty, Kenny Ling, Edward D Wang

**Affiliations:** 1 Medicine, Renaissance School of Medicine, Stony Brook, USA; 2 Orthopaedic Surgery, Stony Brook University, Stony Brook, USA

**Keywords:** atrial fibrilation, medical complications, postoperative complications, propensity score match, total shoulder arthroplasty

## Abstract

Background: Atrial fibrillation (AF) is a common cardiac comorbidity associated with increased risk of bleeding and complications following surgery. Few studies have evaluated the impact of preoperative diagnosis of AF on complications following primary total shoulder arthroplasty (TSA). The purpose of this study is to evaluate the risk of 90-day and two-year postoperative medical and implant-related complications in patients undergoing TSA with a diagnosis of AF.

Methods: A multicentre database, TriNetX (TriNetX, LLC, Cambridge, MA, USA), was queried for patients who underwent primary TSA between 2013 and 2023. After exclusion criteria, patients were divided into two cohorts: those diagnosed with a preoperative diagnosis of AF prior to TSA and those without a preoperative diagnosis of AF (non-AF). The AF and non-AF cohorts were propensity-matched in a 1:1 ratio. Medical complications included sepsis, infection, stroke, pneumonia, renal failure, myocardial infarction (MI), venous thromboembolism (VTE), hemorrhage, blood transfusion, wound dehiscence, superficial surgical site infection (SSI), deep SSI, organ/space SSI, readmission, and mortality. Implant-related complications include revision TSA, periprosthetic joint infection (PJI), periprosthetic fracture, dislocation, and mechanical loosening.

Results: Three thousand eight hundred sixty-four patients were included in each cohort after propensity matching, accounting for any significant differences in patient demographics or comorbidities. Within 90 days following primary TSA, patients with AF showed a significantly increased risk of stroke, pneumonia, and VTE compared to the non-AF cohort (p < 0.05). Within two years following TSA, patients with AF showed a significantly increased risk of stroke, pneumonia, VTE, MI, renal failure, blood transfusion, mortality, total implant-related complication rate, and PJI compared to the non-AF cohort (p < 0.05).

Conclusion: Preoperative diagnosis of AF is associated with an increased risk of 90-day and two-year medical and implant-related complications following primary TSA. Surgeons should be aware of these risks to better optimize their patients.

## Introduction

Total shoulder arthroplasty (TSA), including anatomic and reverse, is primarily indicated for managing severe shoulder joint dysfunctions, including osteoarthritis, rotator cuff arthropathy, and complex proximal humerus fractures. However, postoperative complications occur in approximately 7.4% of cases, encompassing issues such as poor wound healing, infection, and prosthetic dislocation [1,2]. More severe complications include hospital readmission, thromboembolic events, cardiac incidents, and mortality [1-3]. Patient-related risk factors such as chronic medical conditions have been associated with increased risks of both intraoperative and postoperative complications following total shoulder arthroplasty [4]. As projection models in recent years have demonstrated a significant annual increase in incidence of TSA, we can expect the rate of complications to rise as well [5]. Therefore, it is imperative to recognize and address preoperative risk factors to optimize surgical outcomes following shoulder arthroplasty. 

Atrial fibrillation (AF) is a common cardiac arrhythmia that has been evaluated in broader studies of noncardiac surgery, including orthopedic procedures [6-11]. Patients requiring anticoagulation therapy who undergo surgery are inherently at an increased risk for bleeding, which further leads to wound complications, infections, and hematoma formation [11-13]. One particular study evaluated the effect of chronic anticoagulation therapy on patients with AF who underwent total hip or knee arthroplasty. This study found that pre-existing AF was linked to significantly longer hospital stays, increased transfusion requirements, and increased risk of periprosthetic joint infections [11]. Studies addressing the effect of AF in an orthopedic setting have predominantly involved total joint arthroplasty of the hip and knee, rather than TSA. Furthermore, those studies have only focused on short-term complications, readmissions, and length of stay, with little information on long-term prosthesis survival [7-9,11,12]. 

The direct impact of preoperative AF on long-term complications following primary TSA has yet to be well described. This may be an important perioperative consideration, given its rising incidence in the elderly population [14]. The purpose of this study is to evaluate the risk of 90-day and two-year medical and implant-related complications in patients undergoing TSA with a diagnosis of AF. The study aims to compare these patients to a matched control group without AF (non-AF). We hypothesize an elevated risk of both short-term and long-term complications in patients with AF.

## Materials and methods

In this retrospective cohort study, we queried TriNetX, LLC (Cambridge, MA, USA) [15], an aggregated database of deidentified electronic medical records from 49 health-care organizations (HCOs) within the research network, on March 1^st^, 2025, for patients who underwent primary TSA, including both anatomic (aTSA) and reverse TSA (rTSA), from January 1^st^, 2013, to March 1^st^, 2023, allowing for a minimum follow-up of two years. The study was exempt from institutional review board approval due to the use of deidentified patient records.

aTSA and rTSA were analyzed together because TriNetX does not consistently differentiate surgical indications or implant designs across all HCOs. Furthermore, postoperative outcomes are shared across both techniques. Therefore, TSA was evaluated as a combined construct.

A total of 45,120 patients who underwent primary TSA for all indications from 2013 to 2023 were identified using the Current Procedural Terminology (CPT; a standardized procedural coding system maintained by the American Medical Association) code 23472 [16]. Patients younger than 18 years of age at the time of TSA were excluded. Patients with CPT codes indicating revision TSA at index surgery were excluded to avoid misclassification. Patients were then divided into two cohorts. The AF cohort included patients with a confirmed preoperative diagnosis of AF prior to index TSA using the International Classification of Diseases, 10^th^ Revision (ICD-10; a standardized diagnostic coding defined by the Centers for Disease Control and Prevention) code I48.91 (Appendix A) [17]. The non-AF cohort excluded patients without a preoperative diagnosis of AF. Prior to matching, 3,915 patients were in the AF group, and 41,205 patients were in the non-AF group. TriNetX employs automated data quality controls that exclude encounters with missing demographic data and remove duplicate patient records prior to queries [15].

A 1:1 propensity score matching was employed using the TriNetX algorithm, accounting for demographics (age, gender, race, ethnicity) and comorbidities (overweight status, diabetes, hypertension, heart failure, prior transient ischemic attack (TIA) or stroke, and peripheral vascular disease). This resulted in matched cohorts of 3,864 patients each. The platform integrates the nearest-neighbor matching with a tolerance level of 0.01 and ensures that the difference between propensity scores is P ≤ 0.1. Post-matching covariate balance was assessed using standardized mean differences (SMD), with SMD < 0.1 indicating appropriate balance between cohorts, which is consistent with TriNetX validation standards [15].

Outcome variables to assess medical complications included sepsis, infection, stroke, pneumonia, renal failure, myocardial infarction (MI), venous thromboembolism (VTE), hemorrhage, blood transfusion, wound dehiscence, superficial surgical site infection (SSI), deep SSI, organ/space SSI, readmission, and mortality within 90 days and two years postoperatively.

Implant-related outcome variables include revision TSA, periprosthetic joint infection (PJI), periprosthetic fracture, dislocation, and mechanical loosening within 90 days and two years after index TSA. All outcomes of interest were identified using diagnostic ICD-10 codes [17]. All CPT and ICD-10 codes used for case identification, comorbidity assessment, and postoperative outcomes are provided in Appendix A [16,17].

Risk ratios (RR) and 95% confidence intervals (CIs) were computed, and the complication rates were analyzed using the TriNetX system [15]. Categorical variables were assessed using the chi-squared test, while continuous variables were evaluated with independent t-tests. The level of statistical significance was set at P < 0.05.

## Results

Prior to matching, the AF group had a significantly higher mean age compared to the non-AF group, with differences in gender and ethnicity as well. Higher prevalence of comorbidities such as overweight status, diabetes, hypertension, heart failure, prior TIA or stroke, and peripheral vascular disease was also noted to be significant in the AF group (Table 1). After propensity score matching, the demographic and comorbidity variables were no longer statistically significant (Table 1). 

**Table 1:** Patient demographics and comorbidities in patients with and without atrial fibrillation undergoing total shoulder arthroplasty AF, atrial fibrillation; DM, diabetes mellitus; HTN, hypertension; TIA, transient ischemic attack

Characteristic	Unmatched Cohort: AF (% of Cohort)	Unmatched Cohort: Non-AF (% of Cohort)	p-value	Matched Cohort: AF (% of Cohort)	Matched Cohort: Non-AF (% of Cohort)	p-value
Total	3,915 (100%)	41,205 (100%)	—	3,864 (100%)	3,864 (100%)	—
Age at Index	73.20 ± 8.18	68.00 ± 9.57	<0.001	71.70 ± 7.61	71.8 ± 7.60	0.633
Gender						
Male	2,022 (51.65%)	17,212 (41.77%)	<0.001	1,982 (51.29%)	1,985 (51.37%)	0.946
Female	1,735 (44.32%)	22,272 (54.05%)	<0.001	1,725 (44.64%)	1,708 (44.20%)	0.697
Unknown	158 (4.04%)	1,721 (4.18%)	0.673	157 (4.06%)	171 (4.43%)	0.43
Race						
White	3,442 (87.92%)	33,687 (81.76%)	<0.001	3,394 (87.84%)	3,407 (88.17%)	0.649
Black or African American	162 (4.14%)	2,931 (7.11%)	<0.001	162 (4.19%)	159 (4.12%)	0.864
Asian	29 (0.74%)	393 (0.95%)	0.186	29 (0.75%)	26 (0.67%)	0.685
American Indian or Alaska Native	10 (0.26%)	128 (0.31%)	0.55	10 (0.26%)	10 (0.26%)	1
Native Hawaiian or Other Pacific Islander	10 (0.26%)	64 (0.16%)	0.139	10 (0.26%)	10 (0.26%)	1
Other	34 (0.87%)	627 (1.52%)	<0.001	32 (0.83%)	30 (0.78%)	0.799
Unknown	235 (6.00%)	3,375 (8.19%)	<0.001	234 (6.06%)	232 (6.00%)	0.924
Ethnicity						
Hispanic or Latino	74 (1.89%)	1,611 (3.91%)	<0.001	74 (1.92%)	67 (1.73%)	0.552
Not Hispanic or Latino	3,154 (80.56%)	31,366 (76.12%)	<0.001	3,108 (80.44%)	3,101 (80.25%)	0.841
Unknown	687 (17.55%)	8,228 (19.97%)	<0.001	682 (17.65%)	696 (18.01%)	0.677
Overweight and Obesity	870 (22.22%)	5,323 (12.92%)	<0.001	846 (21.89%)	858 (22.21%)	0.742
Type 2 DM	1,001 (25.57%)	6,479 (15.72%)	<0.001	980 (25.36%)	994 (25.73%)	0.715
Primary HTN	2,642 (67.48%)	18,424 (44.72%)	<0.001	2,592 (67.08%)	2,666 (69.00%)	0.107
Tobacco Use	42 (1.07%)	489 (1.19%)	0.528	41 (1.06%)	46 (1.19%)	0.684
Heart Failure	809 (20.66%)	1,274 (3.09%)	<0.001	758 (19.62%)	705 (18.25%)	0.124
TIA or Stroke	233 (5.95%)	823 (2.00%)	<0.001	224 (5.80%)	224 (5.80%)	0.997
Peripheral Vascular Disease	215 (5.49%)	751 (1.82%)	<0.001	200 (5.18%)	195 (5.05%)	0.796

Ninety-day postoperative outcomes 

Compared to the non-AF cohort, patients with AF showed a statistically significant increased risk of stroke (2.5% vs. 1.8%, RR: 1.4, 95% CI: 1.01 to 1.86, z = 2.04, p = 0.041), pneumonia (2.5% vs. 1.7%, RR: 1.5, 95% CI: 1.09 to 2.06, z = 2.48, p = 0.013), and VTE (4.3% vs. 3.2%, RR: 1.3, 95% CI: 1.06 to 1.67, z = 2.46, p = 0.014) within 90 days after undergoing primary TSA (Table 2). However, there were no statistically significant differences in implant-related outcomes within the same follow-up period between the two cohorts (Table 3). 

**Table 2:** Matched cohort comparison of 90-day postoperative medical complications following primary total shoulder arthroplasty AF, atrial fibrillation; VTE, venous thromboembolism; PE, pulmonary embolism; DVT, deep vein thrombosis; SSI, surgical site infection Values in bold indicate statistical significance with the p-value set to <0.05. * Less than 10 instances of the complication were found.

Complication	AF (%)	Non-AF (%)	Risk Ratio	95% CI	p-value
Sepsis	<10* (0.26%)	0 (0.00%)	-	-	-
Infection	22 (0.57%)	16 (0.41%)	1.38	0.99-2.08	0.329
Stroke	96 (2.48%)	70 (1.81%)	1.37	1.01-1.86	0.041
Pneumonia	95 (2.46%)	64 (1.66%)	1.48	1.09-2.06	0.013
Renal Failure	133 (3.44%)	123 (3.18%)	1.08	0.85-1.38	0.525
Myocardial Infarction	26 (0.67%)	15 (0.39%)	1.73	0.92-3.27	0.09
VTE (PE + DVT)	165 (4.27%)	124 (3.21%)	1.33	1.06-1.67	0.014
Hemorrhage	<10* (0.26%)	<10* (0.26%)	1.00	0.42-2.40	1.00
Blood Transfusion	63 (1.63%)	47 (1.22%)	1.34	0.92-1.95	0.124
Wound Dehiscence	<10* (0.26%)	11 (0.29%)	0.91	0.39-2.14	0.827
Superficial SSI	<10* (0.26%)	<10* (0.26%)	1.00	0.42-2.40	1.00
Deep SSI	<10* (0.26%)	<10* (0.26%)	1.00	0.42-2.40	1.00
Organ/Space SSI	0 (0.00%)	0 (0.00%)	-	-	-
Readmission	241 (6.24%)	233 (6.03%)	1.03	0.87-1.23	0.705
Mortality	17 (0.44%)	<10* (0.26%)	1.70	0.78-3.71	0.177

**Table 3:** Matched cohort comparison of two-year postoperative implant complications following primary total shoulder arthroplasty AF, atrial fibrillation; PJI, periprosthetic joint infection Values in bold indicate statistical significance with the p-value set to <0.05.

Complication	AF (%)		Non-AF (%)	Risk Ratio	95% CI	p-value
Total Complication Rate	393 (10.17%)		330 (8.54%)	1.19	1.04-1.37	0.014
Reoperation	120 (1.42%)		117 (1.29%)	1.03	0.80-1.32	0.843
PJI	89 (2.30%)		59 (1.53%)	1.51	1.09-2.09	0.013
Periprosthetic Fracture	34 (0.88%)		40 (1.04%)	0.85	0.54-1.34	0.483
Dislocation	107 (2.77%)		105 (2.72%)	1.02	0.78-1.33	0.889
Mechanical Loosening	33 (0.85%)		37 (0.96%)	0.89	0.56-1.42	0.631

Two-year postoperative outcomes 

Within the two-year postoperative period following TSA, patients with AF still demonstrated a statistically significant increased risk of developing stroke (6.4% vs. 4.7%, RR: 1.4, 95% CI: 1.12 to 1.63, z = 3.18, p = 0.002), pneumonia (9.8% vs. 6.4%, RR: 1.5, 95% CI: 1.31 to 1.78, z = 5.43, p < 0.001), and VTE (8.5% vs. 7.3%, RR: 1.2, 95% CI: 1.01 to 1.36, z = 1.98, p = 0.048) when compared to the non-AF group (Table 4). Additionally, the AF cohort was found to have a statistically significant increased risk of MI (1.3% vs. 0.8%, RR: 1.6, 95% CI: 1.03 to 2.47, z = 2.10, p = 0.036), renal failure (13.8% vs. 10.6%, RR: 1.3, 95% CI: 1.16 to 1.47, z = 4.35, p < 0.001), blood transfusion (4.0% vs. 3.1%, RR: 1.3, 95% CI: 1.02 to 1.64, z = 2.16, p = 0.031), and mortality (2.2% vs. 0.9%, RR: 2.4, 95% CI: 1.60 to 3.51, z = 4.45, p < 0.001) (Table 4). With regards to implant-related complications, patients with AF had a higher statistically significant increased risk of overall complication rate (10.2% vs. 8.5%, RR: 1.2, 95% CI: 1.04 to 1.37, z = 2.46, p = 0.014) and development of PJI (2.3% vs. 1.5%, RR: 1.5, 95% CI: 1.09 to 2.09, z = 2.49, p = 0.013) compared to the non-AF group (Table 5). 

**Table 4:** Matched cohort comparison of two-year postoperative medical complications following primary total shoulder arthroplasty AF, atrial fibrillation; VTE, venous thromboembolism; PE, pulmonary embolism; DVT, deep vein thrombosis; SSI, surgical site infection Values in bold indicate statistical significance with the p-value set to <0.05. * Less than 10 instances of the complication were found.

Complication	AF (%)	Non-AF (%)	Risk Ratio	95% CI	p-value
Sepsis	<10* (0.26%)	0 (0.00%)	-	-	-
Infection	67 (1.73%)	49 (1.27%)	1.37	0.95-1.97	0.092
Stroke	247 (6.39%)	183 (4.74%)	1.35	1.12-1.63	0.002
Pneumonia	377 (9.76%)	247 (6.39%)	1.53	1.31-1.78	<0.001
Renal Failure	533 (13.79%)	408 (10.56%)	1.31	1.16-1.47	<0.001
Myocardial Infarction	51 (1.32%)	32 (0.83%)	1.59	1.03-2.47	0.036
VTE (PE + DVT)	329 (8.51%)	282 (7.30%)	1.17	1.01-1.36	0.048
Hemorrhage	15 (0.39%)	16 (0.41%)	0.94	0.46-1.89	0.857
Blood Transfusion	154 (3.99%)	119 (3.08%)	1.29	1.02-1.64	0.031
Wound Dehiscence	36 (0.93%)	45 (1.17%)	0.80	0.52-1.24	0.315
Superficial SSI	17 (0.44%)	<10* (0.26%)	1.70	0.78-3.71	0.177
Deep SSI	<10* (0.26%)	<10* (0.26%)	1.00	0.42-2.40	1.00
Organ/Space SSI	<10* (0.26%)	<10* (0.26%)	1.00	0.42-2.40	1.00
Readmission	495 (12.81%)	468 (12.11%)	1.06	0.94-1.19	0.352
Mortality	83 (2.15%)	35 (0.91%)	2.37	1.60-3.51	<0.001

**Table 5:** Matched cohort comparison of 90-day postoperative implant complications following primary total shoulder arthroplasty AF, atrial fibrillation; PJI, periprosthetic joint infection Values in bold indicate statistical significance with the p-value set to <0.05. * Less than 10 instances of the complication were found.

Complication	AF (%)		Non-AF (%)	Risk Ratio	95% CI	p-value
Total Complication Rate	152 (3.93%)		131 (3.39%)	1.16	0.92-1.46	0.203
Reoperation	55 (1.42%)		50 (1.29%)	1.1	0.75-1.61	0.623
PJI	32 (0.83%)		23 (0.60%)	1.39	0.82-2.37	0.223
Periprosthetic Fracture	17 (0.44%)		14 (0.36%)	1.21	0.60-2.46	0.589
Dislocation	64 (1.66%)		62 (1.61%)	1.03	1.09-1.78	0.857
Mechanical Loosening	<10* (0.26%)		<10* (0.26%)	1.00	0.42-2.40	1.00

## Discussion

This study investigated the association between preoperative AF and postoperative complications in patients undergoing TSA. Analysis of a large national cohort revealed that patients with AF had significantly higher rates of 90-day complications, including stroke, pneumonia, and VTE. Over a two-year follow-up, AF patients demonstrated an additional increase in risk of MI, renal failure, blood transfusion, and mortality. A higher incidence of total implant-associated complication rate was seen, particularly PJI, in the AF cohort. These findings suggest that AF is an important preoperative risk factor for poor outcomes following TSA. 

The short-term period after total shoulder arthroplasty is important for optimizing recovery, minimizing complications, and establishing long-term implant success [18]. Fox et al. identified preoperative functional status, increasing age, and female gender as significant predictors of postoperative medical complications in an unmatched cohort of 12,982 patients undergoing primary TSA between the years of 2011 and 2016 [19]. Particularly, they reported the incidence of stroke, pneumonia, deep vein thrombosis (DVT), and pulmonary embolism (PE) within 30 days of surgery to be 0.1%, 0.5%, 0.3%, and 0.3%, respectively. In our AF cohort of 3,864 patients, we found the 90-day incidence rate for these medical complications to be significantly higher when compared to the non-AF cohort at 2.5% vs. 1.8% for stroke, 2.5% vs. 1.7% for pneumonia, and 4.3% vs. 3.2% for VTE, and two-year incidence rates to be 6.4 vs. 4.7%, 9.8% vs. 6.4%, and 8.5% vs. 7.3%, respectively. Fox et al. also reported incidence rates of MI, receiving blood transfusion, and mortality within 30 days of primary TSA to be 0.2%, 3.5%, and 0.2%, respectively [19]. Our 2-year analysis evaluating medical outcomes in the AF cohort revealed significantly higher rates when compared to the non-AF cohort: 1.3% vs. 0.8% for MI, 4.0% vs. 3.1% for blood transfusion, and 2.2% vs. 0.9% for mortality. While it is likely that a longer follow-up period could have reflected the overall increase in medical complication rates seen in our study, one study has shown that patients with pre-existing AF carry an increased risk of adverse medical outcomes such as stroke, heart failure, and mortality after non-cardiac surgery [20]. Therefore, our findings can be supported by the notion that AF can amplify the risk of both short-term and long-term medical complications identified in the broader TSA population. This underscores the need for targeted perioperative management and optimization in this high-risk subgroup. 

Renal failure is a significant risk factor for adverse outcomes in patients undergoing TSA, as patients with pre-existing chronic kidney disease (CKD) experience higher rates of perioperative complications, including increased risk of requiring blood transfusion [21]. In our long-term analysis, there was a significant increase in risk of developing renal failure in the AF cohort compared to the non-AF cohort (13.8% vs. 10.6%). Given that AF and CKD frequently coexist and share pathophysiologic mechanisms such as impaired perfusion and neurohormonal dysregulation [22,23], our finding likely suggests that AF has a temporal impact on renal function with a compounded risk profile in TSA patients. Therefore, monitoring renal function is crucial postoperatively. 

Cancienne et al. conducted a similar study using a national insurance database to evaluate postoperative complications in patients undergoing TSA from 2007 to 2016 who required therapeutic anticoagulation for AF compared to those not on anticoagulation [12]. In their cohort of 684 patients who received anticoagulation, they found significantly higher rates of wound infections, including PJI, within both three- and six-month postoperative periods (3.36 vs. 2.12% and 3.95 vs. 2.71%, p < 0.05), respectively [12]. Reoperation rates were also elevated in this group within six months after index TSA (2.49 vs. 1.80%, p = 0.0003) [12]. In contrast, our 90-day and two-year outcomes analysis did not show a statistically significant difference in reoperation rate following TSA. However, we did observe a significantly higher two-year incidence of PJI in the AF cohort compared to the non-AF group (2.3% vs. 1.5%, p = 0.013). AF is well known for its association with chronic systemic inflammation, impaired tissue perfusion, and diminished immune response, all of which can impair wound healing and increase susceptibility to prosthetic joint infections [2,3,12,24-26]. Advanced age, reduced mobility, and greater medical complexity in AF patients may also further contribute to increased infection risk without always requiring reoperation [1]. Although the specific mechanism and associations aren't clearly understood, it would be helpful to further stratify the direct and indirect effects AF has on PJI. It would be interesting to further determine the role of anticoagulation in patients with AF and whether that plays a role in the increased rates of PJI. Nevertheless, our findings highlight the need for further research into the mechanism by which AF affects implant-related complications.

Limitations

This study has a few limitations. First, the retrospective nature of the study hinders the ability to establish causality. The use of a national surgical database limits our ability to account for the duration and type of AF (e.g., paroxysmal vs. persistent), as well as the specific anticoagulation regimen used. As mentioned above, further research may help to determine whether the anticoagulation regimen affects postoperative complication rates. We are also unable to account for any perioperative blood loss or complications, which may skew postoperative outcomes. Combining aTSA and rTSA may introduce variability given the differences in indications and biomechanics. However, TriNetX does not reliably distinguish between these implant types at the procedural coding level across HCOs. Nevertheless, combining TSA approaches is consistent with methodology from prior large national database analyses, and our study focused on complications common to both implant designs. Given that CPT code 23472 captures both aTSA and rTSA and does not permit consistent implant-level differentiation in large administrative datasets, the previous National Surgical Quality Improvement Program and claims-based analyses have evaluated TSA as a combined cohort when assessing postoperative complications and readmissions [19]. Lastly, our analysis was limited to a maximum two-year follow-up period, and thus, longer-term outcomes such as implant loosening or cardiovascular events beyond this time frame were not included. Despite these limitations, our study included a large sample size and used propensity matching of AF and control cohorts to improve the validity of our comparisons. Additionally, data sourced from multiple HCOs enhances the generalizability of our findings. Our findings strengthen current knowledge by providing evidence that AF is an independent risk factor for both short- and long-term systemic and implant-related complications following TSA. 

## Conclusions

Our study identified that a preoperative diagnosis of AF is associated with an increased risk of stroke, pneumonia, and VTE after 90 days following primary TSA and an additional increase in risk of MI, renal failure, blood transfusion, and mortality two years postoperatively. Additionally, the AF patients in our study demonstrated a higher incidence of PJI after two years following TSA. By recognizing the risks associated with preoperative AF, surgeons may be better equipped to identify high-risk surgical candidates and employ risk mitigation strategies such as careful anticoagulation management and cardiac optimization. 

## Appendices

Appendix A 

**Table 6:** CPT and ICD-10 Codes CPT, Current Procedural Terminology; ICD-10, International Classification of Diseases, 10^th^ Revision; TSA, total shoulder arthroplasty; TIA, transient ischemic attack; SSI, surgical site infection; PJI, periprosthetic joint infection Sources: [16,17]

Code Type	Code	Description
CPT	23472	TSA (anatomic or reverse)
CPT	23473	Revision TSA; humeral or glenoid component
CPT	23474	Revision TSA; humeral and glenoid components
ICD-10	I48.91	Atrial fibrillation, unspecified
ICD-10	T84	Complications of internal orthopedic prosthetic devices
ICD-10	Z72.0	Tobacco use
ICD-10	I10	Essential (primary) hypertension
ICD-10	E11	Type 2 diabetes mellitus
ICD-10	I50	Heart failure
ICD-10	Z86.73	Personal history of TIA/cerebral infarction
ICD-10	I73	Peripheral vascular disease
ICD-10	E66	Overweight and obesity
ICD-10	T81.44XA	Sepsis following a procedure
ICD-10	T81.4	Infection following a procedure
ICD-10	I21.9	Acute myocardial infarction, unspecified
ICD-10	I26	Pulmonary embolism
ICD-10	I82	Venous embolism and thrombosis
ICD-10	T84.81	Embolism due to an orthopedic prosthetic device
ICD-10	T84.86XA	Thrombosis due to an orthopedic prosthetic device
ICD-10	J18	Pneumonia, unspecified organism
ICD-10	N17.9	Acute kidney failure, unspecified
ICD-10	I63	Cerebral infarction (stroke)
CPT	36430	Blood transfusion
ICD-10	R99	Ill-defined and unknown cause of mortality
ICD-10	Z48	Encounter for postprocedural aftercare (readmission)
ICD-10	T81.41XA	Superficial incisional SSI
ICD-10	T81.42XA	Deep incisional SSI
ICD-10	T81.43XA	Organ/space SSI
ICD-10	T84.50XA	PJI — unspecified prosthesis
ICD-10	T84.59XA	PJI — other specified prosthesis
ICD-10	T81.31XA	Wound dehiscence
ICD-10	T84.83XA	Hemorrhage due to orthopedic implant
ICD-10	M97.31XA	Periprosthetic fracture — right shoulder
ICD-10	M97.32XA	Periprosthetic fracture — left shoulder
ICD-10	T84.028A	Dislocation of other internal joint prosthesis
ICD-10	T84.029A	Dislocation of unspecified internal joint prosthesis
ICD-10	T84.038A	Mechanical loosening — other prosthetic joint
ICD-10	T84.039A	Mechanical loosening — unspecified prosthetic joint
